# Patellar Malalignment Combined With Deformed Valgus Knee Treated by Distal Femoral Varus Osteotomy and Retrograde Intramedullary Nailing

**DOI:** 10.1111/os.70209

**Published:** 2025-11-21

**Authors:** Chi‐Chuan Wu

**Affiliations:** ^1^ Department of Orthopedic Surgery Chang Gung Memorial Hospital, Chang Gung University Taoyuan City Taiwan

**Keywords:** femur, knee, osteotomy, patellar malalignment, valgus

## Abstract

**Objectives:**

Patellar malalignment (PM) is common and a deformed valgus knee is an uncommon contributing factor. A deformed valgus knee can magnify the traction forces laterally during knee extension, consequently leading to PM. Treatment of combined disorders without correction of the deformed valgus knee is often less effective. The objective of this study was to assess the possibility of using the retrograde intramedullary nailing technique with some modifications for the treatment of both disorders.

**Methods:**

From January 2011 to December 2020, 36 consecutive adult patients with 36 combined disorders underwent surgical treatment. The distal femur was obliquely osteotomized by creating a posterior cam on the distal bony fragment. The cam was consequently inserted into the marrow cavity of the proximal bony fragment. After the bone marrow was reamed, a dynamically locked intramedullary nail was inserted using the retrograde technique. A lateral retinacular release was performed on the patella, and the articular surfaces of the lateral patellar facet and lateral femoral condyle were drilled. The chi‐square test was used to analyze categorical data and the Mann–Whitney U test was used to analyze numerical data.

**Results:**

Thirty‐two patients were followed for an average of 2.8 years (range, 1.7–5.4 years) and all osteotomized sites healed (average, 2.8 months). All 32 deformed valgus knees were corrected to the acceptable axis (from an average of valgus 10° initially to valgus 2° finally in 32 knees on the mechanical axis, *p* < 0.001). All PM had improved congruence angle, lateral patellofemoral angle, and patellofemoral index (*p* < 0.001 in all three). Both the tibiofemoral and patellofemoral joints achieved satisfactory function in all patients (*p* < 0.001).

**Conclusion:**

The described technique can concomitantly treat both the tibiofemoral and patellofemoral joints. Although the technique is relatively simple, the effect is remarkable, and the success rate is high. Therefore, it may be a valuable alternative for the treatment of combined disorders.

## Graphical abstract

Patellar malalignment combined with a deformed valgus knee is treated. After the nail inlet hole is created, the distal femur is obliquely osteotomized by creating a posterior cam on the distal bony fragment. The cam is consequently inserted into the marrow cavity of the proximal bony fragment and the desired axis of the lower extremity is realigned. A dynamic mode of a locked intramedullary nail is retrogradely inserted.

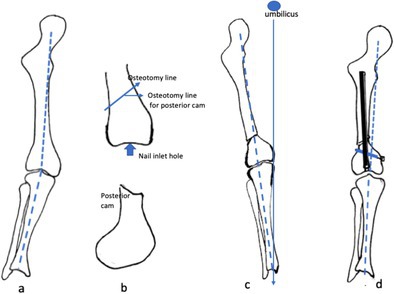

## 1 Introduction

Patellar malalignment (PM) is commonly encountered in orthopedic clinics. An uncommon contributing factor is the deformed valgus knee, which consequently magnifies the quadriceps angle (Q‐angle) and increases the lateral traction forces on the patella [1, 2]. Although PM in most patients is caused by imbalanced peripatellar soft tissue tension and can be successfully treated with nonoperative techniques, PM combined with a deformed valgus knee often requires surgical treatment to restore normal alignment [3, 4]. Surgical indications usually correlate with the severity of valgus knees.

The normal Q‐angle is 11°–13° valgus, which provides outward component forces during knee extension [5, 6]. If the Q‐angle is increased, the outward traction force increases. Once the muscle power of the vastus medialis (especially, the vastus medialis obliquus [VMO]) is lowered, lateral patellar subluxation occurs [7, 8]. Without correction of the magnified knee valgus, treatment techniques are often less effective. In addition, deformed valgus knees cause progressive degeneration in the lateral compartment of the tibiofemoral (TF) joint [9, 10]. Early surgical correction of combined disorders is reasonable.

Following the advancements in modern medicine and technology, techniques for the surgical treatment of both disorders are continuously being developed. A distal femoral or proximal tibial osteotomy may be used to correct knee valgus, each with unique advantages and disadvantages [11, 12]. An intramedullary nail or plate may be used for stabilization of osteotomized fragments [13, 14]. To the best of the author's knowledge, a convincing surgical technique has not been generally acknowledged [11, 15]. In the literature, using the distal femoral varus osteotomy with retrograde intramedullary nail stabilization has been reported successfully in correcting the deformed valgus knee [13]. Theoretically, similar surgical principles may be used to treat both TF and patellofemoral (PF) disorders. This study aimed to assess the possibility of using the retrograde intramedullary nailing technique with some modifications for the treatment of both disorders: (i) Clinically, a plate has been widely used for stabilization after corrective osteotomy. However, a relatively large surgical wound with a relatively high complication rate may occur. (ii) A retrograde intramedullary nail has been used for stabilization by the traditional surgical technique. However, it requires an image intensifier to assist nail or screw insertion. The surgical time may be markedly prolonged. (iii) The osteotomized site may endanger the fracture healing due to defect creation among bone interspaces. With the described modified surgical technique, treatment of such uncommon combined disorders may become more efficient and simpler.

## 2 Materials and Methods

### 2.1 Ethical Declarations

The Institutional Review Board of the author's institution approved this study (IRB: 202400307B0). In accordance with the Declaration of Helsinki, informed consent was unnecessary from all study participants.

### 2.2 Selection of Patients

From January 2011 to December 2020, 36 consecutive patients who had 36 PM combined with deformed valgus knees were surgically treated using the technique described at our institution. A single author performed all operations and followed up all patients. The patients were aged 21–65 years (average, 42 years), with a male‐to‐female ratio of 1 to 5. None of the patients had any evident knee trauma history, and all deformed valgus knees had occurred since youth, with slow progression. Anterior or lateral knee pain occurred intermittently and lasted for several months to years. None of the patients had undergone surgery previously.

At the author's outpatient department (OPD), all patient complaints were examined in detail and the deformed knee was examined carefully. The PF pain was evaluated and confirmed using a grinding test. The anteroposterior, lateral, and Merchant's tangent radiographs, and the full‐length standing scanogram were routinely taken. The indication for surgical correction of the deformed valgus knee was > 5° valgus deformity (i.e., > 5° valgus on the mechanical axis or > 11° valgus on the anatomic axis) [2]. Inclusion criteria of this study were adult patients (> 18 years), > 5° valgus deformity, persistent PF pain (> 3 months with or without physical therapies), with or without positive radiographic findings of PM, and ≤ grade 2 TF osteoarthritis (OA) in the lateral compartment (Ahlback grading classification) [16]. The exclusion criteria of this study were > grade 2 TF‐OA in the lateral compartment, patellar alta, and trochlear dysplasia [17]. Advanced PF‐OA (stage 3 modified Iwano classification) due to long‐term PM was not a contraindication for the described technique [13, 18].

### 2.3 Surgical Technique

**FIGURE 1:**
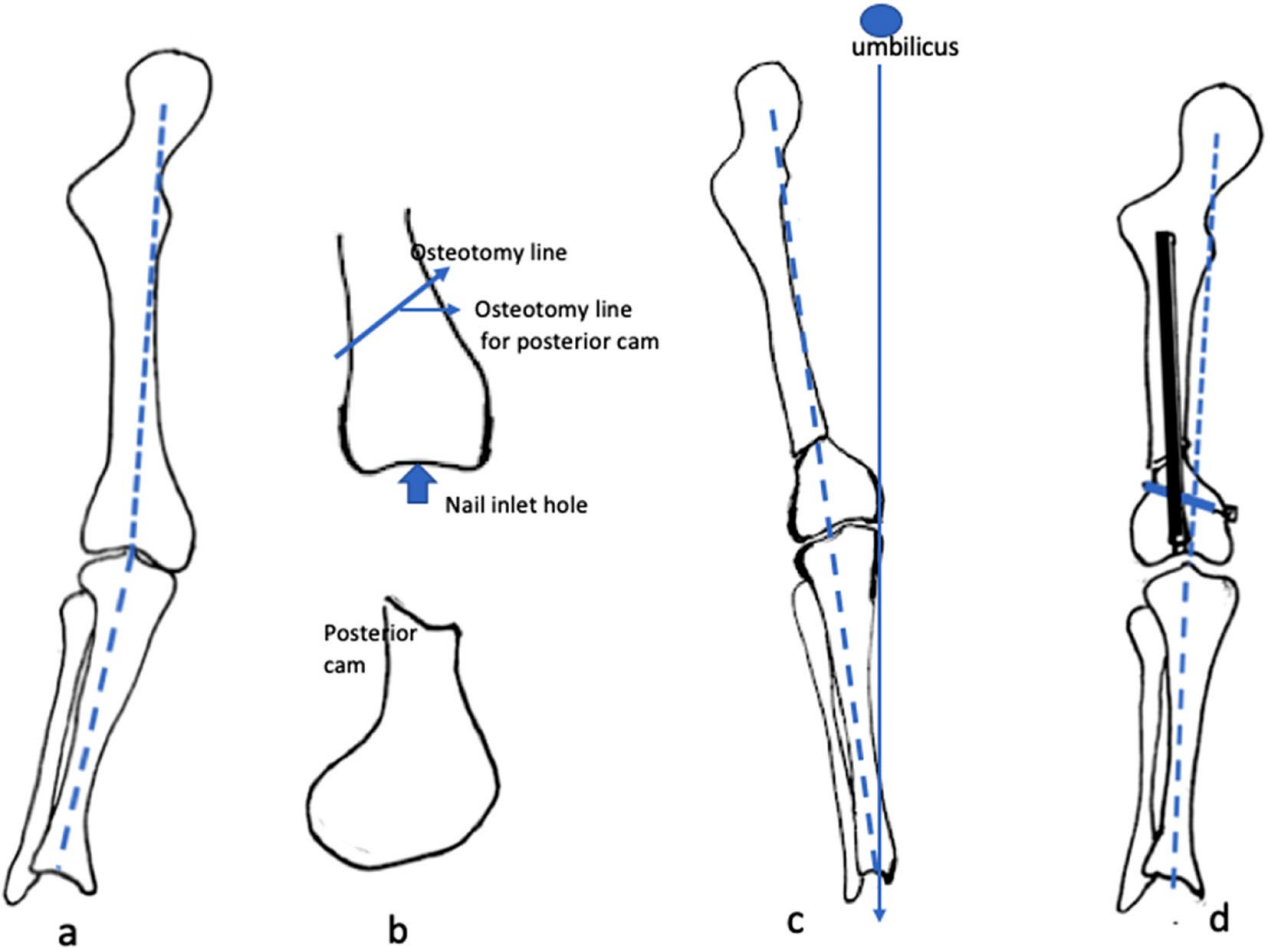
The procedure of the described technique: (a) Patellofemoral malalignment combined with a deformed valgus knee requires operative correction. (b) After the nail inlet hole is created, the distal femur is obliquely osteotomized by creating a posterior cam on the distal bony fragment. (c) The desired lower extremity axis is realigned after the posterior cam of the distal fragment is inserted into the marrow cavity of the proximal fragment. (d) A locked intramedullary nail is inserted with a dynamic mode after intramedullary reaming.

Under general anesthesia with endotracheal intubation or spinal anesthesia, the patient was placed in the supine position on the operating table. A pneumatic tourniquet was routinely used (Figure 1a).

A 5 cm skin incision was made angularly along the inferomedial patella and the medial edge of the patellar tendon. The patella was laterally retracted and the intercondylar notch of the femur was exposed. A 3 mm Kirschner wire was inserted at the center of the trochlear groove, and a flexible reamer was used to create a bony window.

A second 5 cm skin incision was made linearly along the anterolateral thigh over the supracondylar area. With anterior retraction of the vastus lateralis, the femoral supracondyle was well exposed. A 30° upward oblique osteotomy, 3 cm proximal to the upper border of the lateral femoral condyle, was performed using a power saw from the lower lateral cortex to the upper medial cortex. The distal bony fragment was pulled forward by using a bone hook. One centimeter of the anterior cortex of the distal bony fragment was removed using a small power saw, which consequently created a posterior cam (Figure 1b).

The osteotomized fragments were reduced and the cam of the distal fragment was inserted into the marrow cavity of the proximal fragment. The medial edge of the knee was placed in the midline and the axis of the lower extremity was realigned (a line from the umbilicus, the medial edge of the knee, toward a spot 1 cm lateral to the medial malleolus, Figure 1c) [19]. Consequently, a rigid guidewire was inserted through the window of the trochlear groove into the marrow cavity under direct visualization to create a tunnel. The rigid guidewire was then converted to a flexible guidewire and the marrow cavity was reamed as large as possible. Finally, a suitably sized locked intramedullary nail (Russell‐Taylor locked nail, Smith & Nephew Inc., Memphis, TN, USA) was inserted upwards in the dynamic mode (Figure 1d). The local stability of bending and rotation was checked manually. Generally, the stability is sufficient because of the cam effect between fragments.

In patients with combined osteoarthritic PF joints, a lateral retinaculum release was performed on the patella using surgical scissors. The lateral facet of the patella was inverted and the articular surface of the patella was drilled with a 2 mm Kirschner wire (drilling chondroplasty) [18, 20]. The articular surface of the lateral femoral condyle was drilled from the inferomedial aspect of the patella. Finally, the wound was closed with layer‐by‐layer absorbable sutures without drain insertion.

### 2.4 Postoperative Treatment

Postoperatively, knee range of motion exercises were encouraged as early as possible. The patient was allowed to ambulate with partial weight bearing using crutches or a walker. They were followed up at the author's OPD at an interval of 4–6 weeks and the aids were discontinued after the bone healed. Follow‐ups were arranged after 1 year and whenever necessary. At follow‐up, clinical and radiographic healing processes were recorded. The anteroposterior, lateral, Merchant's tangent radiographs were taken. The full‐length standing scanogram was obtained whenever necessary.

### 2.5 Assessment of Treatment

Bony union was defined as the absence of pain and tenderness, the ability to walk without aids, and trabeculae connecting the osteotomized gap radiographically [21]. Nonunion was defined as an osteotomized site that did not heal after 1 year of treatment. Malunion was defined as the healing of an osteotomized site with > 5° valgus deformity.

The knee function was evaluated with the Knee Society Rating System and no less than 80 points was considered satisfactory [22]. The PF joint function was evaluated using the Kujala scoring system, and no less than 80 points was considered satisfactory [23]. Both scoring systems scored 100 points.

### 2.6 Statistical Analysis

SPSS version 20 software (SPSS Inc., Chicago, IL, USA) was used for statistical comparison. *p* < 0.05 was considered statistically significant. The chi‐square test was used to analyze categorical data and the Mann–Whitney U test was used to analyze numerical data.

Sample size was not evaluated in this study. Because this technique had never been reported in the literature before, the least sample size could not be calculated.

## 3 Results

Thirty‐two patients were followed up for at least 1 year (average, 2.8 years; range, 1.7–5.4 years) and all osteotomized sites healed. The union rate was 100% (32/32) and the union time was an average of 2.8 months (range, 2.2–3.6 months). The follow‐up rate was 88.9% (32/36). Four patients could not be contacted despite all efforts.

There were no postoperative complications such as deep wound infection, nonunion, or malunion.

### 3.1 Results After Treatment

Before surgery, 32 patients had deformed knees with an average of 10° valgus (range, 8°–16° valgus) on the mechanical axis. At the latest follow‐up, the knees of 32 patients had an average of 2° valgus (range, 2° varus to 3° valgus) on the mechanical axis (*p* < 0.001, Table 1).

**TABLE 1:** Treatment of combined disorders by the described operative technique (*n* = 32).

Lesion site	Pre‐operative	Latest follow‐up	*p*
Tibiofemoral joint			
Valgus deformity	Average, 10° valgus (range, 8°–16° valgus)	Average, 2° valgus (range, 2° varus to 3° valgus)	< 0.001
Satisfactory function	22%	100%	< 0.001
Radiographical staging			
Grade 0	22%	81%	
Grade 1	50%	19%	
Grade 2	28%	0%	
Patellofemoral joint			
Satisfactory function	37%	100%	< 0.001
Congruence angle	9.4° (5.4°–13.4°)	2.7° (−3.2°–6.4°)	< 0.001
Lateral PF angle	7.9° (2.4°–11.1°)	9.8° (6.4°–11.6°)	< 0.001
PF index	3.6 (2.9–4.3)	1.8 (1.0–2.4)	< 0.001

Abbreviation: PF, patellofemoral.

**FIGURE 2:**
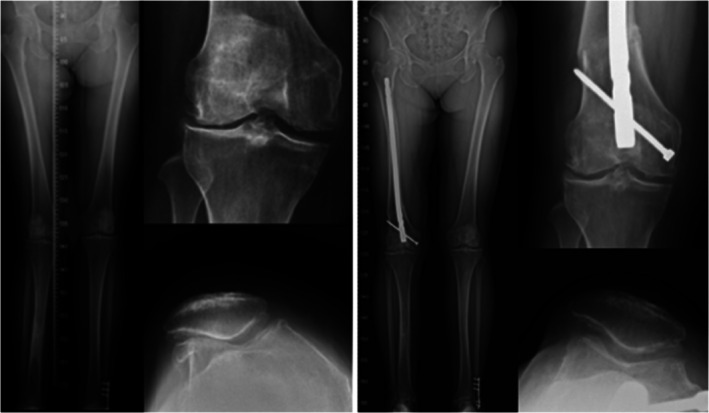
A 48‐year‐old woman sustained right patellofemoral malalignment combined with a deformed valgus knee (left). A distal femoral varus osteotomy with a lateral patellar retinacular release was performed. All anatomic abnormalities with symptoms were recovered for 5.4‐year follow‐up (right).

**FIGURE 3:**
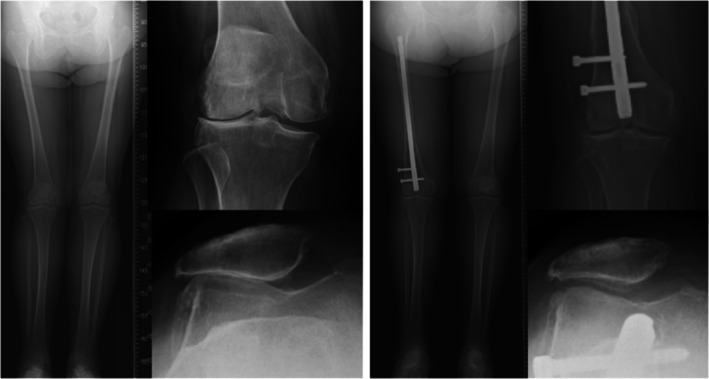
A 57‐year‐old woman sustained right patellofemoral malalignment combined with a deformed valgus knee (left). A distal femoral varus osteotomy with a lateral patellar retinacular release was performed. The tibiofemoral joint improved from grade 2 to grade 0 osteoarthritis and the patellofemoral joint improved from stage 3 to stage 1 osteoarthritis for a 4.2‐year follow‐up (right).

Before surgery in 32 patients, 7 patients (22%) sustained grade 0 TF‐OA. Sixteen and 9 patients had grade 1 and grade 2 TF‐OA, respectively. At the latest follow‐up of 32 patients, 6 patients had grade 1 TF‐OA and the other 26 patients (81%) had grade 0 TF‐OA in the lateral compartment (symmetric joint spaces in both compartments, Figures 2 and 3).

Before surgery in 32 patients, the average congruence angle was 9.4° (range, 5.4°–13.4°), the average lateral PF angle was 7.9° (range, 2.4°–11.1°), and the average PF index was 3.6 (range, 2.9–4.3). At the latest follow‐up of 32 patients, an average congruence angle of 2.7° (range, −3.2°–6.4°, *p* < 0.001), an average lateral PF angle of 9.8° (range, 6.4°–11.6°, *p* < 0.001), and an average PF index of 1.8 (range, 1.0–2.4, *p* < 0.001) were observed. Four patients had stage 3 PF‐OA preoperatively and all were treated and followed up. All patients with stage 3 PF‐OA improved to stage 1 and showed satisfactory function in both joints (Figure 3).

### 3.2 Function After Treatment

The knee function was evaluated in the initial 32 patients, of whom 25 knees were unsatisfactory (78.1%, 25/32). At the latest follow‐up, all 32 patients achieved satisfactory knee function (100%, 32/32, *p* < 0.001).

The PF function was evaluated in the initial 32 patients, and 22 knees were unsatisfactory (62.5%, 20/32). At the latest follow‐up, all 32 patients had achieved satisfactory PF function (100%, 32/32, *p* < 0.001).

## 4 Discussion

The present study reported a valuable surgical technique with a high satisfactory rate, which can concomitantly treat both PM and deformed valgus tibiofemoral joints. Especially, the surgical technique is relatively simple and does not require other auxiliary devices. Theoretically and clinically, it should be worth promoting.

### 4.1 Potential Risks of the Large Q‐Angle

PM is common with an incidence as high as 40% in the normal population [24]. PM is more common than TF disorders in patients without a history of knee trauma. The vast majority of PM cases are caused by imbalanced peripatellar soft tissue tension due to larger outward traction forces on the patella [1, 2]. Anatomically, the Q‐angle provides an outward traction force from knee flexion to extension. If the Q‐angle becomes much larger (e.g., a severely deformed valgus knee), the patella is further pulled laterally over a shallow and wide trochlear groove. Simply reinforcing the inward pulling power by training the vastus medialis is impractical and less effective.

In the present study, a simple surgical technique was developed for the concomitant treatment of combined PM and deformed valgus knees. The outward pulling forces were decreased by lowering the Q‐angle and performing a lateral patellar retinacular release [18]. The injured articular cartilage in the lateral compartment is restored by drilling chondroplasty [18, 20]. The deformed valgus knee was corrected with the varus osteotomy of the femur, and the increased TF stress was released [13]. Therefore, all 32 followed patients achieved satisfactory results in both the TF and PF joints.

Although combined disorders are uncommon, the severity of valgus deformities can cause varying degrees of complaints. If the valgus deformity is not severe (e.g., 5°–10° valgus deformity), various nonsurgical techniques may be attempted first [25]. Once PF pain still persists for more than 3 months, it may be an indication for surgical correction of combined disorders. In this prospective study, we developed a simple surgical technique. The surgical wounds were small (two 5 cm wounds) and the recovery time was short (average, 3 months). In addition, there were few surgical complications, and the satisfaction rate was high (100%).

During surgery, the desired lower extremity alignment is achieved using a simple and reliable technique. In the literature, a convincing mechanical axis was measured using the full‐length standing scanogram [2, 26]. In the present study, acceptable lower extremity alignment was assessed using the described technique (a line from the umbilicus, the medial edge of the knee, toward a spot 1 cm lateral to the medial malleolus, Figure 1c) [19]. Both assessment techniques were evaluated and their correlation was high [19].

Simply performing a lateral patellar retinacular release to treat PM is less effective [27]. This is because this surgical technique can only relieve the outward pulling force exerted by the iliotibial tract. The outward pulling force provided by the magnified Q‐angle cannot be resolved. Correction of the valgus knee to lower the Q‐angle yielded satisfactory results. In the present technique, both outward pulling forces are relieved, resulting in a satisfactory outcome.

### 4.2 Techniques to Vanish Defects Among Bone Interspaces

In this technique, a posterior cam was created on the distal fragment. After the cam is inserted into the marrow cavity of the proximal fragment, local stability is guaranteed to prevent sliding along the osteotomized surface. A different corrective osteotomy was reported previously [13]. The cutting angle is assumed to coordinate with the required correction angle. The technical difficulties often occur during surgery. In the present technique, a precise correction angle is not required to fit the osteotomized site. The final alignment depends on the desired axis; consequently, the surgical technique is simplified (Figure 1c).

In this study, a retrograde locked nail in the dynamic mode was used. Sliding defects in both fragments are prevented by inserting the cam of the distal fragment into the marrow cavity of the proximal fragment. The distal locked screw prevents bending defects in the distal fragment. The femoral isthmus prevents the bending defects in the proximal fragment. The rotational defect is prevented by the femur and nail curvatures and the locked screw. Bone healing is accelerated by the local fragment compression caused by the dynamic mode of a locked nail [28]. Generally, the local stability is sufficient. This can be confirmed by manual testing during the operation.

Various surgical techniques for correcting deformed valgus knees have been published. Common surgical techniques currently include medial closed or lateral opening wedge distal femoral or proximal tibial osteotomies [10, 29]. After the osteotomy, one or two locked plates were used for stabilization [2]. A supplementary lateral patellar retinacular release, drilling chondroplasty, or chondrocyte transplantation may be performed concomitantly. The use of a wedge osteotomy to treat combined disorders has irregularly been reported in the literature [30]. The success rate of a wedge osteotomy in correcting deformed valgus knees is 80%–90% [31]. The present technique has achieved a 100% success rate.

### 4.3 Varied Blocking Screw Surgical Techniques

Blocking screws had been widely used in the treatment of distal femur fractures or corrective osteotomy when a retrograde intramedullary nail was inserted [32, 33]. Although it can greatly help accurate insertion of the nail, several important disadvantages still exist: 1. Accurate insertion of blocking screws in the proximal fragment of the wide marrow cavity requires an image intensifier. Repeating to take images may produce excess irradiation exposure. In the present technique, no image intensifier is used. The lower extremity alignment completely depends on gross checking; 2. Correction of malalignment using a retrograde nail and blocking screws is generally by way of wedged osteotomy. The osteotomized site usually has some gaps, which potentially endanger the fracture healing. In the present technique, the cam of the distal fragment is inserted into the marrow cavity of the proximal fragment, which completely connects both fragments without gaps; 3. The present technique uses a dynamic mode of a retrograde nail. The osteotomized site is compressed, which can enforce the fracture healing and local stability. Especially, a traditional femur or tibial intramedullary nail can be used, which may be widely applied in the world. With blocking screws, the compressive effect may be greatly lowered due to prevention of nail sliding.

Currently, the full‐length standing scanogram is generally acknowledged to be the most practical tool for studying lower extremity alignment [2]. Conceptually, the most convincing mechanical axis is 0° and the anatomic axis is less reliable because of the curvature of the femoral or tibial shaft. It is difficult to define the line used to represent the femoral or tibial shaft [34]. Therefore, in the present study, all assessments of lower extremity angulation depended on the mechanical axis.

### 4.4 Strengths and Limitations

The strengths of the present study may include the following: (1) It was a prospective study with a high follow‐up rate of 88.9% (32/36). All patients were treated and followed up by an identical surgeon. Therefore, this study should be believable without being suspicious. (2) The surgical technique used was cited from related published articles and had been applied for a long period. All fine skills had been improved step by step. In other words, a better principle is created and previous disadvantages were deleted.

The limitations of the present study may include the following: (1) The mechanical axis was used to decide the success or failure of deformity correction. In the literature, the full‐length standing scanogram has been doubted to precisely evaluate the lower extremity axis [26, 35]. However, except for this technique, no other tools can be simple with the acceptable reliability [36]. (2) The follow‐up period was not long (average, 2.8 years). Whether the deformed valgus knee will recur cannot be predicted. Fortunately, the combined disorders have been resolved with satisfactory function. If either disorder recurs, timely conservative treatment can be performed first, and progressive deterioration may be prevented. (3) Several varied techniques have been reported for concomitant treatment of both disorders. However, the long‐term superiority of all proposed techniques could not be compared. Fortunately, the present technique achieved a high success rate, and its performance is worthwhile.

## 5 Conclusion

The author developed a relatively simple and effective surgical technique for the treatment of combined disorders. The surgical wound was small and the implant for stabilization was simple. Patients typically achieve satisfactory outcomes. This may be an excellent alternative for treating combined disorders in the indicated patients.

## Author Contributions

**Chi‐Chuan Wu:** designed the study, collected and analyzed data, and wrote the manuscript.

## Funding

The author has nothing to report.

## Ethics Statement

This study had been approved by the Institutional Review Board (IRB) of the author's institution (IRB: 202400307B0). The requirement for informed consent for participation and publication was waived by the approving IRB. All methods were carried out in accordance with relevant guidelines and regulations.

## Conflicts of Interest

The author declares no conflicts of interest.

## Data Availability

The data that support the findings of this study are available on request from the corresponding author. The data are not publicly available due to privacy or ethical restrictions.

## References

[1] F. B. Imhoff , M. Cotic , F. G. E. Dyrna , et al., “Dynamic Q‐Angle Is Increased in Patients With Chronic Patellofemoral Instability and Correlates Positively With Femoral Torsion,” Knee Surgery, Sports Traumatology, Arthroscopy 29, no. 4 (2021): 1224–1231. 10.1007/s00167-020-06163-6 32683477

[2] S. Taylor and A. Getgood , “Genu Valgum Correction and Biplanar Osteotomies,” Clinics in Sports Medicine 41, no. 1 (2022): 47–63. 34782075 10.1016/j.csm.2021.08.001

[3] S. Farrokhi , B. Meholic , W. N. Chuang , J. A. Gustafson , G. K. Fitzgerald , and S. Tashman , “Altered Frontal and Transverse Plane Tibiofemoral Kinematics and Patellofemoral Malalignments During Downhill Gait in Patients With Mixed Knee Osteoarthritis,” Journal of Biomechanics 48, no. 10 (2015): 1707–1712. 26087880 10.1016/j.jbiomech.2015.05.015 PMC4521908

[4] A. E. Weber , A. Nathani , J. S. Dines , et al., “An Algorithmic Approach to the Management of Recurrent Lateral Patellar Dislocation,” Journal of Bone and Joint Surgery (American Volume) 98, no. 5 (2016): 417–427. 26935465 10.2106/JBJS.O.00354

[5] C. C. Wu , K. M. Yeow , and Y. J. Yeow , “Imaging Approaches for Accurate Determination of the Quadriceps Angle,” Orthopaedic Surgery 12, no. 4 (2020): 1270–1276. 32548902 10.1111/os.12708 PMC7454214

[6] D. A. Abelleyra Lastoria , C. K. Benny , and C. B. Hing , “The Effect of Quadriceps Anatomical Factors on Patellar Stability: A Systematic Review,” Knee 41 (2023): 29–37. 36610240 10.1016/j.knee.2022.12.015

[7] E. Aksahin , O. Kocadal , C. N. Aktekin , et al., “The Effects of the Sagittal Plane Malpositioning of the Patella and Concomitant Quadriceps Hypotrophy on the Patellofemoral Joint: A Finite Element Analysis,” Knee Surgery, Sports Traumatology, Arthroscopy 24, no. 4 (2016): 903–908. 10.1007/s00167-014-3421-7 25398369

[8] X. P. Shui , F. Ye , C. Y. Li , et al., “Effects of Millimeter‐Wave for Preventing Joint Stiffness in the Immobilized Knee Rat Model,” Knee 42 (2023): 236–245. 37086540 10.1016/j.knee.2023.03.019

[9] S. Schroter , C. Konrads , M. Maiotti , et al., “In Closed Wedge Distal Femur Osteotomies for Correction of Valgus Malalignment Overcorrection of mLDFA Should Be Avoided,” Knee Surgery, Sports Traumatology, Arthroscopy 31, no. 9 (2023): 3992–3999. 10.1007/s00167-023-07449-1 37149824

[10] A. N. Berk , K. K. Gachigi , D. P. Trofa , D. P. Piasecki , J. E. Fleischli , and B. M. Saltzman , “Early Postoperative Complications and Associated Variables After High Tibial Osteotomy and Distal Femoral Osteotomy: A 15‐Year Experience From a Single Academic Institution,” American Journal of Sports Medicine 51, no. 10 (2023): 2574–2582. 37417330 10.1177/03635465231183092

[11] G. Puddu , M. Cipolla , G. Ceyullo , V. Franco , and E. Gianni , “Which Osteotomy for a Valgus Knee?,” International Orthopaedics 34, no. 2 (2010): 239–247. 19547972 10.1007/s00264-009-0820-3 PMC2899363

[12] S. L. Sherman , S. F. Thompson , and J. C. F. Clohisy , “Distal Femoral Varus Osteotomy for the Management of Valgus Deformity of the Knee,” Journal of the American Academy of Orthopaedic Surgeons 26, no. 9 (2018): 313–324. 29629916 10.5435/JAAOS-D-16-00179

[13] C. C. Wu , “Retrograde Dynamic Locked Nailing for Valgus Knee Correction: A Revised Technique,” International Orthopaedics 36, no. 6 (2012): 1191–1197. 22307560 10.1007/s00264-012-1495-8 PMC3353075

[14] Y. G. Park , H. Kang , J. K. Song , J. Lee , J. Y. Rho , and S. Choi , “Minimally Invasive Plate Osteosynthesis With Dual Plating for Periprosthetic Distal Femoral Fractures Following Total Knee Arthroplasty,” Journal of Orthopaedic Surgery and Research 16, no. 1 (2021): 433. 34229703 10.1186/s13018-021-02586-0 PMC8259434

[15] H. Peng , A. Ou , X. Huang , et al., “Osteotomy Around the Knee: The Surgical Treatment of Osteoarthritis,” Orthopaedic Surgery 13, no. 5 (2021): 1465–1473. 34110088 10.1111/os.13021 PMC8313165

[16] B. S. Lee , T. H. Kim , S. I. Bin , J. M. Kim , and H. Kim , “Clinicoradiologic Outcomes of Medial Open‐Wedge High‐Tibial Osteotomy Are Equivalent in Bone‐On‐Bone and Non‐Bone‐On‐Bone Medial Osteoarthritis,” Arthroscopy 37, no. 2 (2021): 638–644. 32998040 10.1016/j.arthro.2020.09.033

[17] J. M. Kazley and S. Banerjee , “Classifications in Brief: The Dejour Classification of Trochlear Dysplasia,” Clinical Orthopaedics and Related Research 477, no. 10 (2019): 2380–2386. 31393338 10.1097/CORR.0000000000000886 PMC6999944

[18] C. C. Wu , “Combined Lateral Retinacular Release With Drilling Chondroplasty for Treatment of Patellofemoral Osteoarthritis Associated With Patellar Malalignment in Elderly Patients,” Knee 18, no. 1 (2011): 24–29. 20171107 10.1016/j.knee.2010.01.005

[19] C. C. Wu , “A Novel Approach for Evaluating Acceptable Intra‐Operative Correction of Lower Limb Alignment in Femoral and Tibial Malunion Using the Deviation Angle of the Normal Contralateral Knee,” Knee 21, no. 2 (2014): 573–581. 23195999 10.1016/j.knee.2012.10.011

[20] M. J. Kraeutler , G. M. Aliberti , A. J. Scillia , E. C. McCarty , and M. K. Mulcahey , “Microfracture Versus Drilling of Articular Cartilage Defects: A Systematic Review of the Basic Science Evidence,” Orthopaedic Journal of Sports Medicine 8, no. 8 (2020): 2325967120945313. 32913875 10.1177/2325967120945313 PMC7443991

[21] C. C. Wu , “Aseptic Femoral Nonunion Treated With Exchange Locked Nailing With Intramedullary Augmentation Cancellous Bone Graft,” Journal of Orthopaedic Surgery and Research 17, no. 1 (2022): 339. 35794570 10.1186/s13018-022-03229-8 PMC9258056

[22] J. N. Insall , L. D. Dorr , R. D. Scott , and W. N. Scott , “Rational of the Knee Society Clinical Rating System,” Clinical Orthopaedics and Related Research 248 (1989): 13–14. 2805470

[23] U. M. Kujala , L. H. Jaakkola , S. K. Koskinen , S. Taimela , M. Hurrne , and O. Nelimarkka , “Scoring of Patellofemoral Disorders,” Arthroscopy 9, no. 2 (1993): 159–163. 8461073 10.1016/s0749-8063(05)80366-4

[24] M. A. Rothermich , N. R. Glaviano , J. Li , and J. M. Hart , “Patellofemoral Pain: Epidemiology, Pathophysiology, and Treatment Options,” Clinics in Sports Medicine 34, no. 2 (2015): 313–327. 25818716 10.1016/j.csm.2014.12.011

[25] X. Zhang , J. P. Eyles , J. Makovey , M. J. Williams , and D. J. Hunter , “Is the Effectiveness of Patellofemoral Bracing Modified by Patellofemoral Alignment and Trochlear Morphology?,” BMC Musculoskeletal Disorders 18, no. 1 (2017): 168. 28431578 10.1186/s12891-017-1524-2 PMC5399843

[26] J. Corbett , J. Tai , L. Salmon , and J. Roe , “Comparison of CT and EOS in Assessing Coronal Lower Limb Alignment When Planning Total Knee Arthroplasty,” Knee 42 (2023): 400–408. 37182443 10.1016/j.knee.2023.04.010

[27] V. Drapeau‐Zgoralski , B. Swift , A. Caines , A. Kerrigan , S. Carsen , and M. Pickell , “Lateral Patellar Instability,” Journal of Bone and Joint Surgery (American Volume) 105, no. 5 (2023): 397–409. 36728086 10.2106/JBJS.22.00756

[28] A. Frank , S. Brianza , M. Plecko , M. J. Raschke , and D. Wahnert , “Variable Fixation Technology Provides Rigid as Well as Progressive Dynamic Fixation: A Biomechanical Investigation,” Journal of Bone and Joint Surgery (American Volume) 102, no. 20 (2020): e115. 33086351 10.2106/JBJS.19.01302

[29] S. G. Liu , D. J. Yu , H. Li , et al., “Combination of External Fixation Using Digital Six‐Axis Fixator and Internal Fixation to Treat Severe Complex Knee Deformity,” Journal of Orthopaedic Surgery and Research 18, no. 1 (2023): 65. 36707900 10.1186/s13018-023-03530-0 PMC9881260

[30] J. Fluegel , F. Zimmermann , S. Gebhardt , D. D. Milinkovic , and P. Balcarek , “Combined Distal Femoral Osteotomy and Tibial Tuberosity Distalization Is Effective in Patients Presenting With Patellar Instability and Patellofemoral Pain due to Patella Alta and Femoral Malalignment,” Archives of Orthopaedic and Trauma Surgery 143, no. 5 (2023): 2557–2563. 35861870 10.1007/s00402-022-04541-y

[31] A. Guarino , L. Farinelli , V. Lacono , et al., “Long‐Term Survival and Predictors of Failure of Opening Wedge High Tibial Osteotomy,” Orthopaedic Surgery 15, no. 4 (2023): 1002–1007. 36782306 10.1111/os.13674 PMC10102285

[32] B. Van Dyke , R. Colley , C. Ottomeyer , R. Palmer , and K. Pugh , “Effect of Blocking Screws on Union of Infraisthmal Femur Fractures Stabilized With a Retrograde Intramedullary Nail,” Journal of Orthopaedic Trauma 32, no. 5 (2018): 251–255. 29356801 10.1097/BOT.0000000000001119

[33] M. Kariksiz and O. Karakoyun , “Acute Correction Ofdistal Femoral Deformities by Retrograde Femoral Nail Using Preoperative Planning,” Journal of Orthopaedic Surgery 30, no. 3 (2022): 10225536221143552. 36448519 10.1177/10225536221143552

[34] C. C. Wu , “Integrating Various Common Indexes of Bony Alignment in Femoral Supracondyle,” European Journal of Orthopaedic Surgery and Traumatology 24, no. 7 (2014): 1271–1277. 24292490 10.1007/s00590-013-1373-0

[35] E. J. McWaler , J. Cibere , N. J. Maclntyre , S. Nicolaou , M. Schulzer , and D. R. Wilson , “Relationship Between Varus‐Valgus Alignment and Patellar Kinematics in Individuals With Knee Osteoarthritis,” Journal of Bone and Joint Surgery (American Volume) 89, no. 12 (2007): 2723–2731. 18056505 10.2106/JBJS.F.01016

[36] H. Zhang , Y. Chen , H. Jiang , et al., “Comparison of Accuracy for Hip‐Knee‐Ankle (HKA) Angle by X‐Ray and Knee Motion Analysis System and the Relationships Between HKA and Gait Posture,” BMC Musculoskeletal Disorders 24, no. 1 (2023): 452. 37270561 10.1186/s12891-023-06437-3 PMC10239101

